# Evaluation of Health Economic Loss Due to Particulate Matter Pollution in the Seoul Subway, South Korea

**DOI:** 10.3390/toxics11020113

**Published:** 2023-01-24

**Authors:** Prakash Thangavel, Kyoung Youb Kim, Duckshin Park, Young-Chul Lee

**Affiliations:** 1 Department of BioNano Technology, Gachon University, 1342 Seongnam-daero, Sujeong-gu, Seongnam-si 13120, Gyeonggi-do, Republic of Korea; 2 Department of Mobile IoT, Osan University, 45 Cheonghak-ro, Osan-si 18119, Gyeonggi-do, Republic of Korea; 3 Korea Railroad Research Institute (KRRI), 176 Cheoldobakmulkwan-ro, Uiwang-si 16105, Gyeonggi-do, Republic of Korea

**Keywords:** economic loss, subway PM exposure, health burden, long-term mortality, morbidity

## Abstract

Evaluating an illness’s economic impact is critical for developing and executing appropriate policies. South Korea has mandatory national health insurance in the form of NHIS that provides propitious conditions for assessing the national financial burden of illnesses. The purpose of our study is to provide a comprehensive assessment of the economic impact of PM_2.5_ exposure in the subway and a comparative analysis of cause-specific mortality outcomes based on the prevalent health-risk assessment of the health effect endpoints (chronic obstructive pulmonary disease (COPD), asthma, and ischemic heart disease (IHD)). We used the National Health Insurance database to calculate the healthcare services provided to health-effect endpoints, with at least one primary diagnosis in 2019. Direct costs associated with health aid or medicine, treatment, and indirect costs (calculated based on the productivity loss in health effect endpoint patients, transportation, and caregivers, including morbidity and mortality costs) were both considered. The total cost for the exposed population for these endpoints was estimated to be USD 437 million per year. Medical costs were the largest component (22.08%), followed by loss of productivity and premature death (15.93%) and other costs such as transport and caregiver costs (11.46%). The total incurred costs (per 1000 persons) were accounted to be USD 0.1771 million, USD 0.42 million, and USD 0.8678 million for COPD, Asthma, and IHD, respectively. Given that the economic burden will rise as the prevalence of these diseases rises, it is vital to adopt effective preventative and management methods strategies aimed at the appropriate population.

## 1. Introduction

Exposure to pollution has short- and long-term effects on humans and poses a greater risk to public health than other forms of pollution, such as groundwater contamination or sludge contamination, because it affects more people. Particulate matter (PM) is a complex aerosolized substance produced primarily by vehicle exhausts and road dust. As a result, PM can have a wide range of particle sizes (2.5–10 µM), elemental compositions, and surface areas, producing a variety of health effects on humans [1].

With over eight million daily passengers utilizing the Seoul Metropolitan Subway, subway rail commuting is a major means of transportation in South Korea. However, subway commuters are frequently exposed to indoor PM_2.5_ pollution which is generated inside the subway stations and accumulates in subway platforms, waiting rooms, and train cabins [2]. Elevated PM_2.5_ concentration levels have been reported in subway platforms around the world, including in Los Angeles [3], London [4], Stockholm [5], Budapest [6], and South Korea [1]. PM_2.5_ is a major air pollutant in Seoul’s subway system and has been directly linked to several comorbidities in human health [7,8]. Furthermore, PM_2.5_-bound metals and polycyclic aromatic hydrocarbons (PAHs) have been related to major health effects such as lung cancer and reduced immunological function [9]. Furthermore, because of their high teratogenicity (pre- and early-pregnancy PM_2.5_ exposure has been linked to a higher occurrence of congenital anomalies, exposure to air pollution during pregnancy has, in particular, been linked to the development of congenital abnormalities, low birth weight, stillbirth, and newborn respiratory illnesses) and carcinogenicity [10,11]. Many countries, including the United States, China, the European Union, and India, have included benzo[a]pyrene (B[a]P) and metals in their air quality monitoring standards [12,13]. Because of the subway’s intrinsic characteristics, such as its compact size, airtight atmosphere, and higher passenger density per unit area, great attention has been paid to the interior air quality in subways or subterranean metro stations. Although passengers only spend a brief time in subway stations, repeated exposure to everyday commuters, as well as short-term exposure to excessive levels of air pollutants, can result in severe or acute health impacts. Furthermore, personnel working in subterranean metro stations are exposed to high levels of air pollution for extended periods of time, which might have long-term health repercussions. Although subterranean metro stations have greatly reduced ambient air pollution, internal air quality concerns may be more serious than outside air quality concerns [14,15,16].

South Korean legislation requires the surveillance of varied types of air pollutants, including PM with diameters of 10 µm or less (PM_10_) and 2.5 µm or less (PM_2.5_), nitrogen dioxide (NO_2_), carbon monoxide (CO), sulfur dioxide (SO_2_), and ozone [17]. Particle exposure has been linked to a variety of health issues, including premature death in people with respiratory disease, fatal and non-fatal heart attacks, heart palpitations, asthma, decreased lung function, and increased respiratory symptoms like airway irritation, shortness of breath, or breathing difficulties. In addition to increased cardiovascular morbidity and mortality, recent large-population epidemiological studies have found that PM_2.5_ exposure can contribute to the initiation and progression of diabetes mellitus (DM) as well as adverse birth outcomes [18,19,20,21,22,23,24]. Air pollution causes alteration of the airway epithelial barrier and signal transduction pathways, parenchymal damage, oxidative stress, phagocytosis impairment, inflammatory cell infiltration, dysregulated cell immunity, epigenetic changes, and autophagy [25]. Asthma prevalence, onset, symptoms, and treatment response can all be influenced by air pollution [26]. Air quality is important in the early development of asthma and as a cause of later-life asthma exacerbations. NO2 exposure throughout childhood increases the risk of getting asthma. Exposure to traffic-related air pollution during infancy has been linked to reduced lung function and long-term respiratory consequences in susceptible newborns [27]. PM exposure may produce physiologic changes in the respiratory system. PM suspensions increased cholinergic hyperresponsiveness while decreasing host defense in mice, resulting in neutrophil influx, bronchoalveolar lavage protein, and cytokine release in lung tissues. Ambient air particles may produce reactive oxygen species and inflammatory factors in alveolar macrophages and polymorphonuclear granulocytes [20], and bronchial epithelial cells. Reactive oxygen species, inflammatory factor production, and respiratory inflammation all played important roles in lung tissue destruction and the increased risk of COPD [28,29,30]. The amount of research addressing the processes behind the influence of air pollution on CVD has expanded dramatically over the last decade. The three most common starting mechanisms are (1) oxidative stress and inflammation, (2) autonomic nerve imbalance, and (3) direct particle translocation. These pathways may activate secondary pathways such as endothelial dysfunction, thrombotic pathways, hypothalamic-pituitary-adrenal axis (HPA) activation, and epigenomic changes. The pathways are distinct and occur at different times and locations throughout the body, but they are highly interconnected, with effects that may converge at some point to increase the risk of CVD outcomes [31,32,33]. In 2010, PM_2.5_ was responsible for approximately 7.1% of global mortality. Ambient air pollution is widely established to have a variety of acute and long-term consequences on human health. Several epidemiological studies have found that the amount of particulate matter (PM_2.5_) or NO_2_ in the air is linked to daily mortality, primarily from cardiovascular and respiratory disorders [34,35,36,37]. An epidemiological study by Kim et al. 2017, found considerable deleterious consequences of pollution that endure over the long term. The study discovered that men and the elderly are the most affected, whereas children appear to recover entirely from the early shocks [38]. Kim et al. 2020 and Kim et al. 2022, in a study on the Indonesian population, present evidence of large and long-term harmful consequences of air pollution on mental health. Using a natural experiment in Indonesia, the study discovered that exposure to severe air pollution considerably increases the prevalence of depressive symptoms in both men and women, as well as the incidence of clinical depression in women, even 10 years later. The study also discovered robust substantial effects of pollution on the intensity of moderate symptoms of depression that persist over time in both sexes [39,40]. A study by Jayachandran 2009 on air pollution due to wildfire in Indonesia in late 1997 found that the air pollution led to over 15,500 child, infant, and fetal deaths. The study presented evidence of the most detrimental timing of exposure to pollution in utero that has the greatest influence on survival. Particulate matter has a significant influence on early-life mortality at levels that are common both indoors and outdoors in many poor nations [41].

In 2016, PM_2.5_ was responsible for 4.09 million premature deaths, with chronic obstructive pulmonary disease (COPD), lower respiratory infections, lung cancer, and ischemic heart disease (IHD) accounting for 19.23, 15.97, 6.83, and 38.51% of fatalities, respectively. The health repercussions of PM_2.5_ have a direct influence on economic activity as measured by national accounts and GDP (GDP). Researchers have calculated the economic cost of extra illness and death caused by PM_10_ and 2.5 [42,43,44,45,46]. Kim et al. (2017) evaluated the impact of air pollution on labor supply in the short and long run. Using the 1997 Indonesian forest fires as a natural experiment, the researchers concluded that pollution had long-term detrimental effects on hours worked. Based on their estimates, average pollution levels in 1997, labor force participation rates, and Indonesian minimum wages, a conservative value of roughly USD 10 billion was lost as a result of this pollution incident in the year 2000 alone [47]. The quantification of the impacts of air pollution is becoming an important component in policy formulation. The effects of health on the economy can be seen from the perspectives of the people and the country [48]. As a result, there are two techniques to assess the health costs associated with air pollution: the economic burden of illness assessment and the macroeconomic approach. The former is further subdivided into the human capital approach and the willingness-to-pay technique, whilst the latter is further subdivided into the macro-econometric approach and the general equilibrium approach [49,50]. Several studies link PM exposures to exacerbated chronic illnesses such as COPD and asthma and ischemic heart disease. In this study, we specifically focus on PM_2.5_-associated long-term illnesses, such as ischemic heart disease, and respiratory disorders, such as asthma and COPD [33]. One of the primary goals of this research is to give a complete assessment of the economic impact of PM_2.5_ exposure in the subway, as well as a comparative analysis of cause-specific death outcomes based on the widespread health-risk assessment of the health effect endpoint. The study is based on newly accessible subway PM_2.5_ concentration data as well as an economic assessment of individual health-related expenditures.

## 2. Data and Methodology

### 2.1. Overview

As a prevalence-based technique that calculates the economic cost of all cases in a particular time, we computed the yearly costs related to the health impact endpoints of COPD, asthma, and IHD. The major source for cost estimates was the NHIS claims database, which contained virtually all of Korea’s claim records [51,52]. A person was considered a patient if they had at least one inpatient or outpatient claim that included a diagnosis. We evaluated both direct and indirect expenses in our analyses. Direct costs were those related to diagnosis and medication, whereas indirect costs were those associated with patient and caregiver productivity loss associated with inpatient and outpatient treatment methods. All costs are approximated in USD. The entire population exposed to PM_2.5_ from subway pollution was evaluated to determine the theoretical number of fatalities in the human risk assessment.

### 2.2. Study Area

The Beomgye subway station in Seoul, South Korea, was chosen for the study and was analyzed to determine the PM concentration and human economic loss due to PM_2.5_-induced health damage. The PM levels of Beomgye subway station were obtained from the Korean Railway Research Institute (KRRI).

### 2.3. Data Sources

PM_10&2.5_ levels of Beomgye station for the period January to March 2019 were obtained from the KRRI. The average or mean concentration of PM_10_, PM_2.5,_ and the average number of passengers have been obtained from the KRRI. The 2019 GDP per capita data and total GDP and mortality due to respiratory and circulatory system data were obtained from the Korean Statistical Information Service (KOSIS) [53,54].

**Figure 1:**
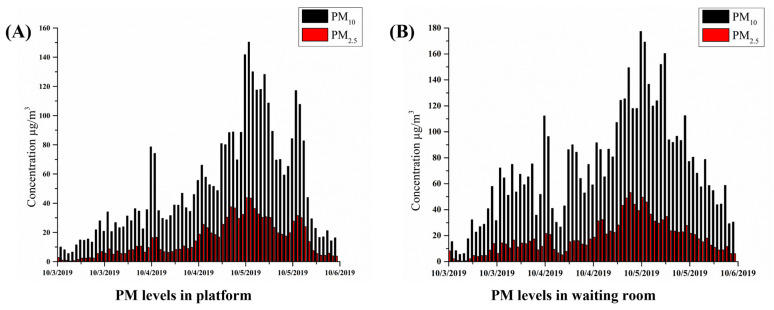
(**A**) PM_10_ and PM_2.5_ concentrations on the platform, (**B**) PM_10_ and PM_2.5_ concentrations in the waiting room.

Daily PM levels of the platform, waiting room, and train cabin, and the number of passengers boarding and getting off the train at Beomgye station, Seoul, South Korea were obtained from the KRRI from January to March 2019 (Table 1). The 24 h data were averaged for all values. The variations in PM_10_ and PM_2.5_ concentrations on the platform and the waiting room are shown in Figure 1.

### 2.4. Exposed Population

Given that air quality is highly connected to the degree of modernization of the transportation system, everyday commuters who utilize the subway are the most likely population exposed to air pollution. As a result, conventional epidemiology considers commuters to be the primary population susceptible to PM_2.5_.

The cabin data of passengers boarding and getting off trains and the data on the total number of people and morbidity and mortality costs (sourced from NHIS) [54,55,56] of each health effect endpoint passengers developing or at risk of PM_2.5_-associated health risks reveal that an average of 30% of passengers will develop PM_2.5_-associated health problems (Table 2). We further normalized to 30% (1000 commuters, 166.6 commuters in a cabin) based on the obtained values of PM_2.5_ concentration and inhaled dose in a cabin linked with PM_2.5_-associated comorbidities, assuming that this 30% of commuters are at risk of developing PM-associated comorbidities.

**Figure 2:**
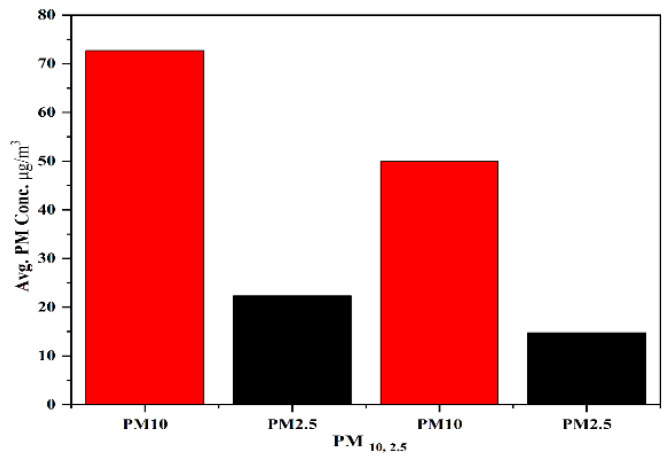
Average concentrations of PM_10_ and PM_2.5_ on the platform and in the waiting room (the two bars on the left represent the values observed in the subway PM and the two bars on the right represent the value defined by WHO.

The GDP per capita, average per capita disposable income, and mortality due to respiratory and circulatory system diseases in Korea in 2019 were sourced from KOSIS and NHIS and were found to be USD 31,846.2, USD 21,882, 36,655, and 60,252, respectively. Moreover, the highest concentrations of PM_10_ and PM_2.5_ were much more than the minimum exposure concentration. The concentrations of PM observed in the subway platforms exceeded the WHO-defined daily values of 50 and 35 μg/m^3^ for PM_10_ and PM_2.5_ [55,56] (Figure 2).

### 2.5. Exposure Route

The human body can be exposed to PM_2.5_ in two ways: through inhalation and eating. PM_2.5_ can directly harm human health by allowing fine particles from the atmosphere to enter the human body through the respiratory tract and travel through the bronchi, disrupting gas exchange in the lungs. Fine dust in the environment can also fall into or settle on food or drink, affecting human health when consumed. To estimate the inhalational or ingested dose of PM_2.5_, we adopted an equation from a study by Manojkumar et al., 2021 [55].

*ID* = *C* × *MV* × *T* (1)

where *ID* is the Inhaled dose per trip (μg), *C* is the exposure concentration (μg/m^3^), *MV* is minute ventilation (m^3^ min^−1^), and *T* is the trip duration (min).

The equation has been widely adopted in previous studies [56,57,58]. In this study, the per-minute ventilation rates recommended by the United States Environmental Protection Agency’s Exposure Factors Handbook, EPA 2011 [59,60] were used and the per-minute ventilation rate for active commuters was set as 0.015 m^3^ min^−1^. Earlier studies adopted similar values for estimating inhaled doses [61,62]. The data are provided in Table 3.

### 2.6. Health-Effect Endpoint

It has been proven that PM_2.5_ can cause severe health risks such as damage to the lungs, respiratory system, and cardiovascular system, increased rates of premature death, and increased risks of cancers, chronic bronchitis, emphysema, and asthma. Based on this causation, we evaluated the economic loss due to PM_2.5_ on the following health effect endpoints:

- COPD (Chronic Obstructive Pulmonary Disease).
- Asthma.
- Cardiovascular diseases.

## 3. Health Economic Accounting

### 3.1. Poisson Regression Model

The likelihood of any endpoint for health effects in the context of the overall population is low, making them low-likelihood events. The actual distribution of endpoints is consistent with the Poisson distribution statistics as a time-series model. The relative risk model of Poisson regression is currently used in health-risk assessments of PM_2.5_ pollution. This works by quantifying the health effect caused by increases in PM_2.5_ concentration by calculating the corresponding health loss. We employed the Poisson regression model since it has been widely adopted by other studies [57].

The formula is as follows:

*E* = exp[*β*(*C* − *C*_0_)] *E*_0_ (2)

Δ*E* = *E* − *E*_0_ (3)

where *E* is the population health effect under the actual concentration of PM_2.5_, *E*_0_ is the population health effect under the PM_2.5_ threshold concentration, *β* is the exposure-response relationship coefficient of a health-effect endpoint, *C* is the mean PM_2.5_ concentration (Mean concentration of platform and waiting room 48.5 μg/m^3^), and *C*_0_ is the threshold concentration of PM_2.5_ (35 μg/m^3^) [53,63]. Δ*E* is the excess health effect—that is, the difference in health effects between the mean concentration and the threshold concentration.

### 3.2. Measurement of Direct and Indirect Cost

#### 3.2.1. Direct Cost

The formal expenses for the diagnosis and treatment of asthma in the official health system were described as direct medical costs and included the costs for inpatient and outpatient care, as well as the costs for any prescription. The care costs assumed by the insurance company were calculated based on the NHIS claims records and KOSIS statistics [63,64,65,66].

#### 3.2.2. Indirect Cost

In general, indirect costs are described as “loss of productivity owing to discontinuation or reduction in productivity due to sickness or death” [59]. In this study, indirect costs were assessed using the human capital method, which assesses human output in terms of market gains and includes sickness costs, death costs, and caregiver time costs (average daily caregiver cost). In the case of patients with severe asthma, the indirect cost was computed by multiplying the number of visits by age and gender, particular average daily income, and employment rates for the age group 20 to 69 years. However, Loss of productivity at work was not considered since the human capital approach is used. Instead, we calculated the total loss of productivity based on (mortality and morbidity) in terms of market gains (GDP). Mortality costs, defined as the loss of potential future income up to the age of 69 due to early death, were calculated by multiplying the amount of associated health effect endpoint by the amount of related health effect endpoint. Deaths in 2019 with gender- and age-specific average yearly earnings and employment rates for each death were used to calculate the present value of future admissions using a 3% discount rate. The expenses of caregiving time were calculated using literature studies and NHIS claims. In Korea, a family member, generally a middle-aged woman, provides practically all care for each health effect endpoint. To measure both direct and indirect costs, we employed the scheme of Lee et al., 2011 [63].

The scheme for estimating indirect costs was as follows:

- Morbidity costs
- Mortality costs
- Caregivers’ time costs
- Methods used to determine the health care costs are given in Table 4

##### Morbidity Costs

(4) $$=\underset{j}{\sum }\left\{\left(I_{ij}+1/3O_{ij}\right)\right\}D_{ij}E_{ij}$$

where *I* is the age, *j* is the gender, *I* is the total number of inpatient days, *O* is the number of outpatient visits, *D* is the average daily earnings, and *E* is the employment rate.

##### Mortality Costs

(5) $$\begin{array}{c} =\underset{i}{\sum }\underset{j}{\sum }\underset{t}{\sum }\left[\frac{F_{ij}r_{jj}^{t+k}E_{ij}}{{\left(1+r\right)}^{k}}\right] \end{array}$$

where *I* is the age, *j* is the gender, *t* is the age of death, *F* is the number of deaths, *γ* is the average yearly productivity at *t* *k* (The exponent “*t* + *k* denotes the adjusted years of productivity loss due to premature death before the age of 70), *E* is the employment rate, *k* is the number of years after death until the age of 70 (average years of life lost before the age of 70), and *r* is the discount rate. For each health effect endpoint, the number of years of life lost correlates with productivity loss.

##### Caregivers’ Time Costs

(6) $$\frac{\sum_{i}\sum_{j}\left(N_{ij}\times IC\right)}{E_{j}\sum_{i\geq 10}\left(0_{ij}\times IC\right)1/3}$$

where *I* is the age, *j* is the gender, *N_ij_* is the number of days spent in the hospital, *O_ij_* is the number of outpatient visits, and *IC* is the average daily informal care expenditures. The caregiving expenses were determined by dividing the average daily caregiving cost by the total days of hospital admissions obtained from the NHIS [42].

## 4. Results and Discussion

The changes in PM concentrations are characterized by the tallest column in Figure 1. The highest concentrations of PM_10_ and PM_2.5_ on the platform and in the waiting room were 150.38 and 43.24 μg/m^3^, and 177.27 and 53.24 μg/m^3^, respectively, and the lowest were 20.34 and 4.32 μg/m^3^, and 5.55 and 0.34 μg/m^3^, respectively (Figure 1A,B). The concentrations also peaked from midday to midnight, indicating that temperature, ventilation, and seasonal variation might play a major role in PM concentrations inside the subway. As shown in Table 3, the inhaled doses estimated using the above formula were 7.2, 14.5, and 21.8 µg for PM_2.5_ for 30, 60, and 90 min trips, respectively. Total costs were calculated according to each component of direct and indirect costs. We applied the Poisson regression model to estimate the risk of each health effect endpoint. To calculate a per capita cost, the anticipated total expenditures were divided by the number of commuters at risk of acquiring PM_2.5_ comorbidities for each health consequence endpoint. The data collected were then statistically analyzed. The economic cost of each health effect endpoint was measured using Equations (1)–(6). Using mortality and morbidity estimations and the economic costs for each health effect endpoint, we calculated the total economic loss for the exposed population developing PM_2.5_-related health effects (Table 5).

In this study, we analyzed the PM_2.5_ levels over a period of 3 months (January-March) in Beomgye station, Seoul, Korea. The PM_2.5_ levels found in the subway, on the platform, and in the waiting room exceed the defined values set by WHO, which in turn poses a great risk to the commuters and the operators. People with chronic disease of the lung, COPD, and asthma, and the aged are at higher risk of developing further complications. On our comprehensive health risk assessment of each health effect endpoint, we estimated that 373 (COPD), 44 (Asthma), and 19 (IHD) commuters (per trip in a train) were at risk of developing PM_2.5_-mediated comorbidities for each health effect endpoint. The total cost incurred by these comorbidities was estimated to be USD 437 million annually (Table 5). Medical costs were the largest component (22.08%), followed by lost productivity and premature deaths (15.93%) and nonmedical costs such as transportation and caregiver costs (11.46%) (Table 6). The total costs incurred by health effect endpoints (per 1000 persons) were estimated to be USD 0.1771 million (COPD; Table 6), USD 0.42 million (Asthma; Table 6), and USD 0.8678 million (IHD; Table 6). The finding that medical charges (direct costs) accounted for the majority of the overall cost suggests that PM_2.5_-induced health consequences are burdensome chronic conditions that need extensive outpatient monitoring. This pattern, which has been observed in other developed nations, might be considered an indirect indicator of a health consequence endpoint being adequately managed at the outpatient level and not progressing to the emergency or hospitalization level [67]. The lost productivity, premature deaths, and transportation and caregiver cost ranked second due to subjective assumptions, such as children needing medical attention for PM_2.5_-induced health effects, as other studies have established [68,69]. The heaviest economic burden is for commuters aged above 60 because of their high mortality and morbidity and this burden is mainly driven by the high proportion of hospitalization and inpatient treatment. Nonetheless, to evaluate the true indirect costs, productivity loss in any form must be considered [70,71,72,73]. As a result, the true socioeconomic burden in South Korea, including the low productivity due to health effect endpoints, will be much higher than our estimates. In addition, a previous study determined that workplace inactivity accounted for 50% of total productivity losses; as this factor was considered based on the KOSIS statistics, we have not considered actual productivity loss at the workplace in our study, and the real socioeconomic costs of each health endpoint may be more substantial than those reported in this study. Our findings indicated that the health burden and economic losses associated with short-term air pollution exposure are significant [74]. To date, only a few studies on the health burden caused by short-term exposure to air pollutants have been conducted in China, Korea, and other countries. The exposure-response coefficients of short-term exposure to air pollutants are likely to be much lower than those of long-term exposure [4,75,76].

In recent years, PM_2.5_ has been widely regarded as the primary air pollutant with the greatest health impact. Several studies have reported that the concentrations of PM_2.5_ inside trains are generally lower than in subway stations, which suggests that time spent on the platform and in the waiting room can be a better predictor of personal exposure [76,77]. However, commuters who travel every day are repeatedly exposed, which can lead to chronic health effects. Nonetheless, even short periods spent in underground environments can exacerbate health risks for vulnerable groups such as children, the elderly, and individuals with pre-existing health conditions. Train operators and other employees who spend many hours in the subway each day are more likely to be exposed to PM_2.5_ levels than the public and are thus at a potentially greater health risk.

A number of studies have revealed that high traffic counts are connected with an increased incidence of respiratory disease [78]. One large British assessment on traffic-related pollution discovered a risk gradient that rose significantly with day-to-day exposure [79]. A large study in southern California found an increased incidence of asthma and wheezing among children who commute on a daily basis [80]. Another study, conducted by Jerrett et al. using data from the same southern California cohort, found a connection between the incidence of asthma and other illnesses and exposure to traffic-related pollution [81]. Much remains unknown about which contaminants cause the majority of respiratory impacts and which signaling pathways are important for exacerbating chronic respiratory disorders. The processes underlying the induction of respiratory aggravation and ischemic heart disease by subway particles require further exploration, as do the interactions of coarse subway particles with major pollutants in provoking oncogenesis.

## 5. Limitations and Conclusions

The limitation of our approach to study was that work engagement was not taken into account when evaluating indirect costs, and because we utilized GDP per capita, the loss of work engagement cannot be depended upon. Cost-of-illness studies, including this one, fail to account for job involvement because existing report sets do not gather this information on a regular basis; instead, such information is often collected through self-reporting [82]. Implied speculations regarding lost production, productive time, and caretaker qualities may compromise the accuracy of projected indirect costs. The methodology for evaluating indirect costs in cost-of-illness research studies is organically based on various assumptions about human and productivity values; it is crucial to compare research with similar assumptions and methodologies [83]. The findings of this study come from a relatively small proportion of the population (1000); this may have led to an underestimation of outcomes. Another limitation of this study is that there is disagreement over the validity of NHIS claims data in terms of diagnostic accuracy. According to one research, only 70% of discharged diagnoses in NHIS claims data were concordant with medical records [84,85]. By using the direct costs of inpatient and outpatient care and indirect costs composed of morbidity and mortality costs based on NHIS claims and KOSIS, the outcomes may vary from those of other similar studies [86,87].

Furthermore, the results provided here are based on PM_2.5_, but no correlation is made between more toxic particles, such as those with high levels of sorbed toxic components. The study ignores the effects of exposure to ultrafine particles, which are implicated in numerous diseases, but for which measurements are lacking.

Altogether, the study found that the economic burden of COPD, Asthma, and IHD caused by commuter subway exposure to PM_2.5_ is large, with significant shares of overall expenditures ascribed to direct and indirect expenses. Given that the burden would increase in tandem with increases in these illnesses, effective preventive and management measures targeting the right population are necessary.

## Figures and Tables

**Table 1:** Average concentrations of PM_10_ and PM_2.5_ and average number of patients traveled.

Platform μg/m^3^		Waiting Room μg/m^3^		Cabin μg/m^3^		Avg. No. of People Travelling per Day	
PM_10_	PM_2.5_	PM_10_	PM_2.5_	PM_10_	PM_2.5_	Boarding	Alighting
42.18	21.00	8.74	5.01	20.69	13.13	5478	5364
83.90	23.72	56.14	18.17	32.64	24.40	8331.6	8388
92.04	22.46	72.84	21.17	49.82	41.05	7306.2	7089
72.70	22.38	49.9	14.23	34.383	26.19	7038.6	6947

**Table 2:** Burden of PM_2.5_ per 1000 persons for each health effect endpoint.

Disease	Risk/Prevalence/Development of a Disease per 1000 Patients (Morbidity)	Mortality-Associated Costs (Million USD) per 1000 Persons	Average Median PM_2.5_ Exposure Concentration (μg)
COPD	373	9.3	1.05
Asthma	44	1.1	1.03
IHD	19	0.4	1.07

**Table 3:** Average dose inhaled per trip at different time intervals.

Average Time Spent by a Commuter in the Subway (min)	30	60	90
Average exposure concentration PM_2.5_ (µg/m^3^)	16.22	16.22	16.22
Average minute ventilation rate by commuter (m^3^ min^−1^)	0.015	0.015	0.015
Inhaled dose per trip (μg) PM_2.5_	7.299	14.598	21.895

**Table 4:** Methods used to determine health care costs.

Health Care Costs	Outpatient	Inpatient	Formula
**Direct medical costs**	✓	✓	The NHIS database was used to get hospital expenses covered by co-payment plans in Korea for inpatients and outpatient visits, including Emergency Department visits.
**Nonmedical costs**	✓	✓	Total transportation expenses were determined by multiplying transportation costs by the number of outpatient visits and hospitalizations based on NHIS claims for each kind of hospital treatment. The overall caregiving expenses were estimated by dividing the average daily caregiving cost by the total days of hospital admissions obtained from the NHIS. Using the human capital method, productivity costs for patients under 70 and over 60 years old were assessed.
**Transportation**			
**Caregiving**		✓	
**Indirect costs**	✓	✓	

**Table 5:** Direct and indirect costs of each disease.

Disease	Direct Cost (Million USD; per 1000 Persons)	Indirect Cost (Million USD; per 1000 Persons)	Total Cost (Million USD; per 1000 Persons)
COPD	0.10591	0.0718	0.17771
ASTHMA	0.55	0.07	0.42
IHD	0.57478	0.294	0.86878

**Table 6:** Economic loss due to COPD, asthma, and IHD.

Category	Costs (Million USD; per 1000 Persons)					
	COPD	Contribution (%)	Asthma	Contribution (%)	IHD	Contribution (%)
Direct						
Medical						
Formal (Treatment)	0.029	16.6	0.2	54.8	0.302	34.76
Informal (Medical Equipment)	0.00631	3.5	0.02	7.4	0.16	18.41
Non-Medical						
Transportation	0.0006	0.3	0.08	2.4	0.07	8.066
Nursing	0.070	39	0.05	14.1	0.04278	4.92
Indirect						3.33
Loss of Work	0.0588	32.7	0.04	12.4	0.029	30.5
Premature deaths	0.013	7.7	0.03	9	0.265	100
Total	0.17771	100	0.42	100	0.86878	

## Data Availability

Not applicable.
